# Creating a Canadian Indigenous Research Network Against Cancer to Address Indigenous Cancer Disparities

**DOI:** 10.1200/JGO.19.00049

**Published:** 2020-01-13

**Authors:** Angeline Letendre, Gail Garvey, Alexandra King, Malcolm King, Reg Crowshoe, Lea Bill, Nadine R. Caron, Brenda Elias

**Affiliations:** ^1^ Alberta Health Services, Edmonton, Alberta, Canada; ^2^ Menzies School of Health Research, Brisbane, Queensland, Australia; ^3^ University of Saskatchewan, Saskatoon, Saskatchewan, Canada; ^4^ Piikani First Nation, Alberta, Canada; ^5^ Alberta First Nation Information Governance Centre, Calgary, Alberta, Canada; ^6^ University of British Columbia, Vancouver, British Columbia, Canada; ^7^ University of Manitoba, Winnipeg, Manitoba, Canada

## Abstract

**PURPOSE:**

In Canada, Indigenous peoples’ cancer rates have increased, but cancer screening rates tend to be lower. When coupled with poor cancer prognosis, treatment barriers, and inaccessible health care, Indigenous patients with cancer experience many unmet needs. Further complicating their journey is a multijurisdictional system that complicates cancer control services, treatments, patient supports, and cancer surveillance. To address these issues, the Canadian Indigenous Research Network Against Cancer (CIRNAC) was developed. This article describes the forerunners and consultative process that created the network and the consensus model developed to ground this network with, by, and for Indigenous peoples.

**METHODS:**

A consultative workshop was held to (1) establish and increase network membership, (2) enhance partnerships with Indigenous communities and other researchers, and (3) develop an Indigenous-led research program, new funding, and related initiatives.

**RESULTS:**

Participants viewed the CIRNAC as a reflective parallel network led by Indigenous peoples that would identify research priorities within Canada, assess how these priorities align with Indigenous patients’ cancer care and research needs, and cross-check to see if these priorities align with each other. The network would also advocate for Indigenous elders/knowledge holders and community grassroot processes to drive research and training, thus demonstrating the power of the community voice and lived experience in research. In addition, the network would foster research partnerships to investigate alternative Indigenous models for cancer prevention, care, treatment, and support.

**CONCLUSION:**

The CIRNAC evolved as a viable vehicle to address cancer with, for, and by Indigenous peoples. The network is guided by a preamble, a set of aims, and an inclusion engagement circle model. It is evolving through major world initiatives, with the aim of formally becoming an internationally linked national network.

**CONTEXT**

**Key Objective**
To describe the context and processes that led to the development of the Canadian Indigenous Research Network Against Cancer.
**Knowledge Generated**
This paper makes transparent the processes and foundational knowledge required to create a network dedicated to advancing Indigenous leadership in cancer research.
**Relevance**
This paper is novel by making transparent the dynamics of creating a network to advance Indigenous leadership in cancer research. As a result, this work can inform the evolution of other networks dedicated to advancing Indigenous health and wellbeing.

## INTRODUCTION

First Nations, Métis, and inuit peoples are the founding Indigenous peoples of Canada. According to the 2016 Statistics Canada census, 1,673,785 Indigenous peoples (First Nations, 977,230; Métis, 587,545; Inuit, 65,025; other Indigenous, 43,935) account for 4.9% of the total Canadian population. More than one half (56.8%) of First Nations peoples reside in the western provinces. Nearly two thirds of Métis live in metropolitan areas, and 80% live in Ontario and the western provinces. Approximately three quarters (72.8%) of the Inuit live in the Inuit Nunangat territory (north). Although the Indigenous population is a significantly younger population, Indigenous peoples 65 years of age and older now account for a larger share.^1^

In Canada, cancer affects mostly Canadians 50 years of age and older, but it can occur at any age (pediatric to adults). Cancer incidence varies as a result of risk factors, and cancer deaths diverge because of incidence, early detection practices, and differential access to screening, diagnosis, treatment and follow-up.^2^ For Canadian Indigenous peoples, increasing cancer rates, poor cancer prognoses, treatment barriers, and inaccessible health care suggest that Indigenous patients with cancer have many unmet needs.^3-8^ Cancer screening rates are lower, and it is unclear why screening is not widely available.^6-10^ Although advanced treatment and care planning options are available in the Canadian health care system, it is not clear how cancer service agencies are incorporating these options for Indigenous populations.^4,6-8^ Moreover, there is a disconnect in Canada between the way service providers think of and direct the structure and delivery of services for Indigenous peoples and the way Indigenous peoples approach and respond to service providers and available treatment.^6-8,11,12^ Erratic reporting of the Indigenous population’s cancer burden in Canada also continues, thus revealing a significant Indigenous health measurement gap, which is shared globally.^13-16^

These barriers, challenges, and gaps in Canada’s cancer care services for Indigenous peoples are further complicated by a complex jurisdictional landscape.^6-8^ Canadian provinces have a constituted responsibility for health services. The federal government has a constituted fiscal responsibility for Status First Nations and Inuit (eg, fiscal transfers, tax arrangements, negotiated resource revenue sharing), and the Canadian Charter of Rights and Freedoms acknowledges First Nation, Inuit, and Métis peoples’ right to self-government.^17^ Given these barriers, gaps, and challenges and the multijurisdictional complexity, Canada needs an Indigenous population–led research agenda to understand cancer prevention, care continuum, surveillance and reporting, and lived experience. In response, this article describes a process to create a Canadian Indigenous Research Network Against Cancer (CIRNAC) to develop and lead this agenda.

## METHODS

In 2016, six authors of this article were invited to present at the inaugural World Indigenous Cancer Conference (WICC) in Brisbane, Australia, by the Australian Indigenous conference hosts (G.G. as lead). On the final day, Canada was selected to host the second WICC. On returning to Canada, the team formed a small western network (n = 9) of Indigenous medical professionals, policy and program directors, elders/knowledge holders, and Indigenous and non-Indigenous health researchers. Only one person in the network was non-Indigenous. All team members were recognized for their cancer research and for working with Indigenous peoples experiencing cancer. To evolve and ground this network, the following methods were used.

Drawing from the Australian National Indigenous Cancer Network^18^ development processes, the inaugural CIRNAC members submitted a successful research workshop planning grant to the Canadian Institutes of Health Research Institute of Indigenous Peoples’ Health and received additional funding from the Canadian Institutes of Health Research Institute of Cancer Research and the Canadian Partnership Against Cancer. For this workshop, the team developed a 3-day face-to-face meeting agenda for participants to:

1. Create a CIRNAC mission and vision statement;

2. Identify research partnership models;

3. Generate critical research priorities to address gaps and identify assets and strength-based approaches; and

4. Consider potential knowledge mobilization approaches, evaluation planning, and training for high-quality research trainees (community and academic).

Evaluation planning was later dropped by workshop participants because it was considered premature. The workshop agenda outlined six key stakeholder presentations, followed by roundtable discussions and breakaway groups with note-takers to document the discussions. The team generated a list of potential workshop invitees (ie, stakeholders) thematically grouped as Indigenous academic, community-based researcher, government and nongovernment organization representative, elder/knowledge holder, and funder. Using a snowball approach, invitees were asked to propose other participants. The workshop was attended by 25 participants, of whom 22 were Indigenous. Funders and government representatives opted to participate with observer status. All participants, however, had an equal voice and contributed supportive ideas. At workshop end, results were summarized and circulated to participants for comment, and feedback was incorporated into a final report.

## RESULTS

### Why a CIRNAC?

A common theme across presentations focused on why a network was needed. The first narrative described the evolution of the Australian National Indigenous Cancer Network and how it led to the WICC and the World Indigenous Cancer Network. The second narrative reported on the increased cancer burden among Canada’s Indigenous peoples, the data gaps, and the need for an Indigenous population–led research network grounded in Indigenous peoples’ knowledge/ceremony, framed by Indigenous ethical principles and connected to Indigenous peoples’ lived experience with cancer. A third narrative highlighted the need to improve Indigenous patients’ cancer data and their availability. A fourth highlighted why Indigenous peoples’ ways of knowing were key to pathways to living well and respect for Indigenous philosophies of health and healing. Elders/knowledge holders, Lea Bill and Reg Crowshoe, then presented on the value of embracing Indigenous life and worldviews and the merit of recognizing and transcending worldviews to avoid cultural confusion. They highlighted an ethical window through which two world views may interact and offer an opportunity for knowledge holders and researchers to collaborate, learn, and share ethical space.^19^ Participants then provided their observations, which thematically framed the network.

### Framing the Collective How of the CIRNAC

First and foremost, authentic community engagement was key. A CIRNAC must be led by Indigenous academics, community members, leaders, and people with lived experience. Elders/knowledge holders were viewed as fundamental guides in network research activities and Indigenous research. Any proposed or developed research would require community readiness and researcher awareness of those communities. Researchers should not assume that all communities know about cancer and its pathways. At the heart of community engagement should be an openness to benefiting the community. Although these approaches have multiple ethical and engagement layers, researchers would learn about the value and role of protocol and ceremony in their research via strong partnerships with elders/knowledge holders.

### Valuing Relationships, Partnerships, and Roles in CIRNAC

Another theme was the collective value of reciprocal relationships and partnerships. One lesson shared came from a First Nation, federal, and provincial partnership that showed collaboration as having a greater impact than statistics alone. For example, although statistics can provide a cancer profile of First Nations people, using stories via a support booklet to accompany statistical reports would emphasize cultural safety and humility, traditional wellness, relationship-based care, and shared decision making for Indigenous patients with cancer, families, and communities. Another lesson was that partners need to reciprocally understand Western and Indigenous viewpoints and fully support cancer care and research for Indigenous peoples. Also required were community-based protocols that include elders/knowledge holder engagement, Indigenous peoples’ ceremonies, and relationship building. For example, respectfully engaging elders/knowledge holders and valuing traditional medicine and ceremonies would lead to holistic care and family inclusion. Understanding rural and urban living differences would lead to initiatives that bring treatment closer to home. Customized cultural safety training programs, research protocol training, and governance agreements for Indigenous peoples would advance Indigenous-led surveillance and health reporting. Respectful negotiations, a strong working relationship, acknowledging and creating safe and ethical space, providing sustained support, and sharing a common goal could decrease Indigenous cancer disparities. After this discussion, breakout groups identified the following roles for a CIRNAC.

### Role 1: Acknowledging and Advocating an Ethical Space Window

A key role of the CIRNAC would involve advocating for a window between two cultures that acts as a form of ethical space.^19^ This space would provide an opportunity to look into the window of another culture and build further understanding of these worldviews. In that space, there would be a multitude of possibilities to identify parallel systems, both Western and Indigenous, which would work toward improving Indigenous peoples’ health and well-being. Via this role, CIRNAC would advocate and defend the need to create that ethical space and educate researchers on how to engage and advocate for the inclusion of Indigenous knowledge systems in cancer research, care, and service delivery. The CIRNAC would also develop advocacy mobilization tools, which when combined with health literacy, could teach about ethical space viewed through multiple lenses. These tools would be distinction based, reflecting diverse cultural and knowledge systems among First Nations, Métis, and Inuit communities, thus demonstrating the inappropriateness of single advocacy approaches.

### Role 2: Promoting Engagement as a Reciprocal Relationship

The network would be grassroots based, facilitating the creation of ethical space between Indigenous communities, researchers, and various agencies. It would help identify peer research associates in communities and across academic and agency settings. It would provide a safe space to support Indigenous researchers who feel caught between two worlds. It would foster an ethical and safe space by bringing together people from different worldviews without the subjugation of one worldview or knowledge system over the other. Through this space, researchers would be supported and inspired to generate knowledge, share information, and translate knowledge for community-led action. In short, the CIRNAC should aim to advocate for a reciprocal two-way process, whereby community, agency, and academic perspectives are shared to foster insight by each party.

### Role 3: Addressing Information Poverty Environments of Indigenous Communities

The CIRNAC would also work toward ending information data poverty by advocating for national data and regional registries, capacity in analysis and interpretation, and Indigenous data stewardship and governance protocols. To ensure positive use of data, the CIRNAC would lead the development of a course for researchers to learn about cultural safety through the lens of Indigenous peoples. This course would engender a responsibility to continue learning about Indigenous contexts and to apply that learning when analyzing and interpreting data. Researchers and the affiliated medical community would learn how to engage communities and appreciate community perspectives on cancer. Communities would learn how to use data with their stories about cancer. For such research and engagement to occur, the CIRNAC would work with funders to support community-based research. It would also foster working groups to help solve data access challenges and safely resolve issues pertaining to Indigenous identifiers for data disaggregation.

### Role 4: Engaging Actionable Knowledge

In addition, the CIRNAC would foster research on how to apply respectful engagement practices to address prevention and care gaps. To advocate for change, research and surveillance is needed to document disparities, identify prevention and care gaps or initiatives that work, and investigate whether guidelines are inclusive, fair, and appropriate for particular populations. To create and engage actionable knowledge, the CIRNAC would therefore facilitate the space to improve prevention, screening, and care for Indigenous peoples.

### Role 5: Honoring Traditional Knowledge, Philosophies of Wellness, and Elders/Knowledge Holders

The CIRNAC would also create an ethical space for elders/knowledge holders.^19^ In return, elders/knowledge holders would facilitate ethical spaces to promote cross-cultural understanding and the value of traditional Indigenous medicines and foods for improved cancer outcomes. Elders/knowledge holders would also play a key role in illuminating and validating an Indigenous person’s lens through thoughtful reflection, storytelling, and ceremony.

### Developing the CIRNAC Model

At workshop end, participants viewed the CIRNAC as a reflective parallel network, whereby Indigenous peoples and their allies would mobilize and advocate in a multitude of ways. Second, it was critical for the network to operationalize Indigenous control and leadership in cancer with full accountability to grassroots Indigenous peoples. Third, the network would be ideal to identify cancer research priorities, evaluate how these priorities align with Indigenous cancer care and research needs, and cross-check to see if these priorities align with other countries (eg, via the World Indigenous Cancer Network). Fourth, the network would also advocate for Indigenous grassroots processes to drive research and create educational opportunities for community and academic researchers. The network would demonstrate by design, presence, and action the power of the community voice and lived experience in research. Fifth, network members would mobilize and advance community learning and develop opportunities for Indigenous academic researchers. Sixth, the network would create and foster research partnerships to investigate alternative Indigenous models for cancer prevention, care, treatment, and support. Finally, elders/knowledge holders must be an integral part of the network to lead access to traditional knowledge, identify disruptions to traditional lifestyles that affect wellness, and show through story-telling, ceremony, and song what we can learn from past experiences.

As a collective, participants agreed that the CIRNAC was a critical resource to improve cancer outcomes with, by, and for Indigenous peoples. With this agreement secured, participants developed the following preamble to guide the network:

The following concepts, understandings, ways of knowing and doing are foundational to a research network on cancer in First Nations, Inuit and Métis: ethical space, oral knowledge, natural laws, Indigenous worldviews and knowledge systems, Indigenous practices and standards, upholding the relationality of all within the universe and our ongoing relationships with each other.

We acknowledge the diversity of Indigenous peoples. We are committed to respectfully engage with Indigenous leadership, to respect and include the knowledge and capabilities of Indigenous people with cancer experience, to collaborate with Indigenous researchers and health professionals and non-Indigenous allies, to work with cancer care and support services, cancer agencies, policy and decision-makers, to provide ethical spaces for Indigenous oral practices, standards and ways of doing, to respect healing and engagement through cultural translations, and to be inclusive of parallel thinking in health systems.

The spirit of the document is to articulate the worldviews, concepts and ideas of Indigenous peoples’ in broadening perspectives on research against cancer to better advance and focus resources on Indigenous research priorities.

The Network is inclusive of students and practitioners of Indigenous and Western research, elders/knowledge holders, Indigenous people, their communities and leadership.

The following overarching network aims were then agreed on: (1) Recognize, acknowledge, gain a better understanding of, advocate for, and respect the lived experiences of Indigenous peoples with cancer and their families. (2) Attest to the importance of wellness through the interrelationships among food, land, medicine, and ceremony and through ways to respect their interrelationships in living a good life and advancing wellness throughout the cancer journey. (3) Honor and connect the diverse lived experiences of Indigenous peoples across the cancer journey, and to strengthen and uphold the capacity of Indigenous peoples experiencing cancer, their families, supports, communities, and organizations in self-determining ways to improve holistic research processes and impacts. (4) Improve cancer outcomes and wellness throughout the lifespan for Indigenous peoples, their families, and their communities; this includes facilitating primary, secondary, and tertiary prevention of cancer, as well as palliative care. (5) Support mentorship and training activities related to Indigenous cancer research and knowledge use at the academic and community levels and to support two-way education, whereby community and academia can learn from each other; and (6) support the creation of an elders/knowledge holders council and the development of a declaration (elders’ statement) to guide the network, with explicit and implicit recognition of the four dimensions of wellness—physical, mental, emotional, and spiritual. This work would build on the Alberta First Nations Elders’ Declaration on Health, which addresses the Truth and Reconciliation Commission Calls to Action and the United Nations Declaration on the Rights of Indigenous Peoples.

**FIG 1:**
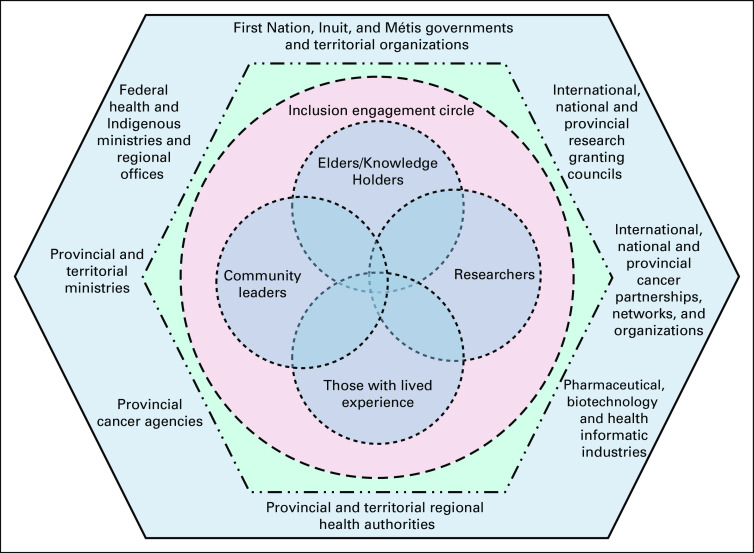
Canadian Indigenous Research Network Against Cancer (CIRNAC) inclusion engagement circle within Canada’s health service and funding system.

The CIRNAC inclusion engagement model was then developed (Fig 1) to illustrate a way for network members to work together. Fundamental to this model was an inclusion circle to support linkages among elders and knowledge holders, those with lived experience, community leaders, and researchers as partnered allies. For these linkages to occur, however, there must be authentic engagement and knowledge mobilization (Western and Indigenous) that advances respectful understanding.

Via this engagement circle, network members would advance ethical models of research that include research as ceremony, inclusive advocacy processes, knowledge of communities and cultural groups, respectful relationships, and engagement of elders/knowledge holders for guidance, engagement, and navigating oral structures (eg, stories and songs). By doing so, network members would be committed to drive change within Canada’s complex health service and funding system, elevate community benefits, avoid harms, develop business models that are respectful of Indigenous peoples, incorporate foundational Indigenous principles into their research, engage reciprocity and develop partnerships, establish and maintain a positive cultural knowledge of cancer, promote Indigenous-led publications and ownership of knowledge, advance decolonization practices, support community-led research, build community research capacity, share power and resources, and expand Indigenous wellness perspectives. To resolve issues, network members would also draw on the principles of reconciliation as determined by, and to a large extent defined by, elders/knowledge holders.

## DISCUSSION

This article shows how the CIRNAC evolved before and through this workshop process. After the workshop, with no administrative support or funding for face-to-face meetings, the core group relied on e-mail communications, Web-based meetings, and leveraged opportunities to meet face to face. Web-based meetings were effective in maintaining communications, whereas face-to-face meetings fostered more collaboration and creativity. The CIRNAC has since evolved by hosting the 2019 WICC in Calgary, Alberta, Canada. For this event, the core group received financial support from the Canadian Partnership Against Cancer, the Canadian Institutes of Health Research, the Alberta Health Services (Canada), and the Alberta First Nations Information Governance Centre (Canada). Via this opportunity, we developed a governance and sustainability structure—an executive committee of Indigenous and non-Indigenous academics, funders, and nongovernmental organization representatives, and an elders/knowledge holder committee/council with representation from Canada’s First Peoples. Also developed were a scientific committee and an international committee. Each committee has a chair and a co-chair. All committees are overseen by an executive chair (director) supported by a coordinator. In addition, we developed an interim financial system and sponsorship list. To achieve sustainability, the CIRNAC aims to formalize partnerships with Indigenous provincial/territorial organizations, federal-provincial-territorial governments, cancer agencies, regional health authorities, industry, cancer foundations, and research granting councils. A postconference event (2019) is scheduled to discuss potential network membership, partnerships, sponsorship, communications, and evaluation.

## Data Availability

The following represents disclosure information provided by authors of this manuscript. All relationships are considered compensated unless otherwise noted. Relationships are self-held unless noted. I = Immediate Family Member, Inst = My Institution. Relationships may not relate to the subject matter of this manuscript. For more information about ASCO's conflict of interest policy, please refer to www.asco.org/rwc or ascopubs.org/jgo/site/misc/authors.html. Open Payments is a public database containing information reported by companies about payments made to US-licensed physicians (Open Payments). No potential conflicts of interest were reported.

## References

[1] Statistics Canada Aboriginal peoples in Canada: Key results from the 2016 Census https://www150.statcan.gc.ca/n1/en/daily-quotidien/171025/dq171025a-eng.pdf?st=P1ivQ-ZF

[2] Government of Canada, Canadian Cancer Society Canadian cancer statistics: A 2018 special report on cancer incidence by stage http://www.cancer.ca/~/media/cancer.ca/CW/cancer%20information/cancer%20101/Canadian%20cancer%20statistics/Canadian-Cancer-Statistics-2018-EN.pdf?la=en

[3] Mazereeuw MV Withrow DR Diane Nishri E et al Cancer incidence among First Nations adults in Canada: Follow-up of the 1991 Census Mortality Cohort (1992-2009) Can J Public Health 109 700 709 2018 29981110 10.17269/s41997-018-0091-0 PMC6964591

[4] Withrow DR Pole JD Nishri ED et al Cancer survival disparities between First Nation and non-Aboriginal adults in Canada: Follow-up of the 1991 census mortality cohort Cancer Epidemiol Biomarkers Prev 26 145 151 2017 27965294 10.1158/1055-9965.EPI-16-0706

[5] Mazereeuw MV Withrow DR Nishri ED et al Cancer incidence and survival among Métis adults in Canada: Results from the Canadian census follow-up cohort (1992-2009) CMAJ 190 E320 E326 2018 29555862 10.1503/cmaj.170272 PMC5860893

[6] Canadian Partnership Against Cancer First Nations cancer control in Canada baseline report. https://www.partnershipagainstcancer.ca/wp-content/uploads/2017/12/first-nations-cancer-control-baseline-report.pdf

[7] Canadian Partnership Against Cancer Métis cancer control in Canada baseline report. https://www.partnershipagainstcancer.ca/wp-content/uploads/2017/12/metis-cancer-control-baseline-report.pdf

[8] Canadian Partnership Against Cancer Inuit cancer control in Canada baseline report. https://www.partnershipagainstcancer.ca/wp-content/uploads/2017/12/inuit-cancer-control-baseline-report.pdf

[9] Elias B Kliewer EV Hall M et al The burden of cancer risk in Canada’s indigenous population: A comparative study of known risks in a Canadian region Int J Gen Med 4 699 709 2011 22069372 10.2147/IJGM.S24292 PMC3206113

[10] Mazereeuw MV Yurkiewich A Jamal S et al Cancer risk factors and screening in First Nations in Ontario [in English, French] Health Promot Chronic Dis Prev Can 37 186 193 2017 28614046 10.24095/hpcdp.37.6.02 PMC5650013

[11] Fuchsia Howard A Smillie K Turnbull K et al Access to medical and supportive care for rural and remote cancer survivors in northern British Columbia J Rural Health 30 311 321 2014 24483272 10.1111/jrh.12064

[12] Poudrier J Mac-Lean RT ‘We’ve fallen into the cracks’: Aboriginal women’s experiences with breast cancer through photovoice Nurs Inq 16 306 317 2009 19906281 10.1111/j.1440-1800.2008.00432.x

[13] Moore SP Antoni S Colquhoun A et al Cancer incidence in indigenous people in Australia, New Zealand, Canada, and the USA: A comparative population-based study Lancet Oncol 16 1483 1492 2015 26476758 10.1016/S1470-2045(15)00232-6

[14] Sarfati D Robson B Equitable cancer control: Better data needed for indigenous people Lancet Oncol 16 1442 1444 2015 26476759 10.1016/S1470-2045(15)00295-8

[15] Elias B Busby K Martens P One little, too little: Counting Canada’s indigenous people for improved health reporting Soc Sci Med 138 179 186 2015 26112164 10.1016/j.socscimed.2015.06.014

[16] Sarfati D Garvey G Robson B et al Measuring cancer in indigenous populations Ann Epidemiol 28 335 342 2018 29503062 10.1016/j.annepidem.2018.02.005

[17] Greenwood M de Leeuw S Lindsay N Challenges in health equity for Indigenous peoples in Canada Lancet 391 1645 1648 2018 29483024 10.1016/S0140-6736(18)30177-6

[18] National Indigenous Cancer Network Improving outcomes for Indigenous people with cancer http://www.nican.info

[19] Ermine W The ethical space of engagement Indig Law J Univ Tor Fac Law 6 193 203 2009

